# Factors predicting cardiovascular events in chronic kidney disease patients. Role of subclinical atheromatosis extent assessed by vascular ultrasound

**DOI:** 10.1371/journal.pone.0186665

**Published:** 2017-10-18

**Authors:** José M. Valdivielso, Angels Betriu, Montserrat Martinez-Alonso, David Arroyo, Marcelino Bermudez-Lopez, Elvira Fernandez, for the NEFRONA investigators

**Affiliations:** 1 Vascular and Renal Translational Research Group, UDETMA, REDinREN del ISCIII, IRBLleida, Lleida, Spain; 2 Statistics Department, University of Lleida, Lleida, Spain; Nagoya University, JAPAN

## Abstract

Patients with chronic kidney disease (CKD) have an increased incidence of cardiovascular events (CVE). The contribution of subclinical atheromatosis extent, including femoral arteries, to CVE in CKD patients has not been investigated. In this paper, we examine the prognostic value of subclinical atheromatosis extent, assessed as the number of arterial territories with plaque, in predicting the incidence of major and minor CVE. The NEFRONA is a multicenter, prospective cohorts study that recruited 2445 CKD subjects and 559 controls, free from previous cardiovascular disease, in 81 medical centers across Spain. The presence of atheroma plaque was assessed by arterial ultrasound in ten arterial territories (carotid and femoral). The predictive power of the presence or absence of atheroma plaque in any territory was compared with the quantification of atheroma extent as the number of territories with plaque. During the median follow up of 48 months, 216 CVE were reported. Factors predicting the incidence of CVE in the whole cohort were being male, CKD patient, lower levels of 25(OH) vitamin D, higher levels of cholesterol and the extent of subclinical atheromatosis, yielding a higher concordance (C) index than the presence or absence of plaque. In stratified analysis including specific factors of CKD patients not on dialysis, the variables predicting CVE were the same as in the whole cohort, plus higher levels of potassium. Again, the inclusion of the information about atheromatosis as number of territories with plaque, presented a higher C index than the presence or absence of plaque. In the dialysis population, significant variables were older age, diabetes, dialysis vintage and higher levels of cholesterol and phosphate. In this case the higher C index was obtained with the information about plaque presence.

Subclinical atheromatosis extent, including femoral arteries, influences CVE in CKD and its detection could improve the prediction of cardiovascular events.

## Introduction

Cardiovascular disease is the main cause of death in chronic kidney disease (CKD) patients, in which cardiovascular death is a more likely outcome than progression to end-stage renal disease (ESRD).[1] Important advances have been made in the knowledge of specific cardiovascular risk factors in CKD. However, neither traditional risk factors, risk score charts for general population nor emerging risk biomarkers appear to significantly increase the prediction power in this population.[2–4] Thus, classical risk prediction equations underestimate cardiovascular disease risk in adults with CKD [5, 6] and new tools for risk prediction in renal patients are urgently needed.

The contribution of atheromatous disease in cardiovascular mortality of CKD patients has not been clearly defined. This can be explained by the fact that in dialysis patients most of the cardiovascular deaths are attributed to sudden death. However, recent data reveal that these events, although triggered by electrolyte disorders, are also influenced by heart remodeling and microischemia of the myocardium.[7] Indeed, the presence of carotid atheroma plaque was found to be independently associated to having a cardiovascular event in dialysis patients.[8, 9] In contrast to dialysis patients, in the CKD population not in dialysis, atherothrombotic deaths are very common,[10] and atherosclerosis prevalence is higher than in the general population.[11] In this case, no studies have evaluated the contribution of atheroma plaque to cardiovascular mortality.

Arterial ultrasonography assessment of subclinical atheromatosis is a non-invasive imaging technique that could predict the incidence of cardiovascular events in CKD patients.[12, 13] The presence of atheroma plaque has been proven to be helpful in cardiovascular risk stratification in the general population and in dialysis patients.[8, 14] The increase of predictive power using the extent of atheromatosis, and the location of the atheroma plaques is also currently under investigation. Historically the assessment of vascular atherosclerosis has been made in carotid arteries. This is due to the fact that several studies have shown that carotid plaque predicts the presence of coronary artery disease.[15] However, the assessment of atherosclerotic plaques in other accessible territories like the femoral arteries is a new field of study. In a post-mortem study in the Netherlands, the femoral artery was the artery most frequently affected by atherosclerosis among 5 peripheral vascular sites, including the common carotid artery.[16] Furthermore, in two very recent studies in middle aged man, association with risk factors and positive coronary disease was stronger in femoral than carotid arteries.[17] Furthermore, in the NEFRONA population, it was reported to be a 13% of CKD patients and a 10% of controls with atheroma plaque exclusively in the femoral arteries.[11] Thus, when patients are screened exclusively in the carotid arteries, an important percentage can be underdiagnosed. Therefore, a combination of carotid and femoral plaque assessment could improve cardiovascular risk prediction.

To this day, no studies have investigated the predictive role of the atheroma extent on cardiovascular risk incidence of CKD patients. The NEFRONA study was specifically designed to determine the predictive accuracy of the number of arterial territories with atheroma plaque in the cardiovascular risk of a cohort of CKD patients after 4 years of follow up. In this paper we present the analysis of cardiovascular events incidence, in order to assess the predictive power of atheroma extent compared with a simpler option like the presence or absence of atheromatosis in any territory.

## Materials and methods

The protocol of the study was approved by the ethics committee of the Hospital Universitario Arnau de Vilanova (Lleida) and all patients were included after signing informed consent. This research followed the principles of the Declaration of Helsinki. The NEFRONA is an observational, prospective, multicentric, cohorts study that aimed to assess the role of the detection of subclinical atheromatosis extent by arterial ultrasound in the cardiovascular risk prediction of CKD patients. The design and objectives of the NEFRONA study have been published in detail.[18, 19] Briefly, 3004 CKD patients and controls (559 individuals with no CKD, 950 in CKD stage 3, 807 in stage 4–5 and 688 in dialysis) who were 18–75 years of age, free from previous cardiovascular events, were enrolled from 81 Spanish hospitals and primary care facilities between October 2009 and June 2011 and followed for at least 4 years to register cardiovascular events.

### Clinical data and laboratory examinations

Current health status, medical history, former cardiovascular risk factors and drug use information was obtained at baseline. A physical examination was performed, consisting of anthropometric measures, standard vital tests and ABI measurements as previously described.[11] Biochemical data were obtained from a routine fasting blood test performed three months either before or after the vascular study. Glomerular filtration rate (GFR) was estimated using the Modification of Diet in Renal Disease Study formula (MDRD-4). Parathyroid hormone (PTH) levels in dialysis patients were corrected using a well-established method to avoid inter-method variability between different centers.[20] The levels of hsCRP and 25(OH) vitamin D were both analyzed in a centralized laboratory; hsCRP was determined by immunoturbidimetric method (Roche/Hitachi modular analytics) and 25(OH) vitamin D levels by Elisa (IDS, UK).

### Ultrasound imaging

B-mode ultrasound of the carotid and femoral arteries was performed using the Vivid BT09 apparatus (General Electric) equipped with a 6–13 MHz broadband linear array probe as previously described.[21] IMT was measured in all 10 regions and calculated as the average between left and right sides. The value of IMT in a region with plaque was truncated to 1.5 mm. The presence of atheromatous plaques was defined as IMT > 1.5 mm protruding into the lumen, according to the ASE Consensus Statement[22] and the Mannheim cIMT Consensus.[23]

An atheromatous plaque presence analysis was performed in 10 territories (both internal, bulb and common carotids, and both common and superficial femoral arteries) by a single reader in blinded fashion, using semi-automatic software (EchoPAC Dimension, General Electric Healthcare). To assess intra-observer reliability, a sample of 20 individuals was measured 3–5 times on different days. A kappa coefficient of 1 was obtained for plaque assessment, indicating excellent intra-observer reliability. The reader was unaware of patients’ clinical histories.

ABI was defined as the ratio between systolic blood pressure in posterior tibial or dorsalis pedis arteries, divided by the highest systolic blood pressure value of both brachial arteries, all of them detected with a continuous Doppler probe. The modified method was chosen for a higher sensitivity, selecting in each patient the most pathological of the four values.[24] A pathologic ABI was defined as a value ≤0.9 (diagnostic of limb ischemia) or ≥1.4 (related to arterial incompressibility and stiffness, usually ascribed to vascular ageing degeneration).

### Follow up

A 4 year follow-up (with bi-annual questionaries’ recordings) was performed by the refererring physician collecting data on cardiovascular events (both fatal and non-fatal). Furthermore, the change in medications and the occurrence of non-cardiovascular death or the start of renal replacement therapy was reported every six months. The cardiovascular events were defined according to the International Classification of Diseases, Ninth Revision, Clinical Modification (ICD9-CM) which includes unstable angina, myocardial infarction, transient ischemic attack, cerebrovascular accident, congestive heart failure, arrhythmia, PAD or amputation for vascular disease and aortic aneurism. Cardiovascular mortality causes including myocardial infarction, arrhythmia, congestive heart failure, stroke, aneurism, mesenteric infarction, and sudden death. Importantly, CVD events and deaths were accurately recorded by each physician responsible of the recruitment of patients. In the case of an out-of-hospital death, family members were interviewed by telephone to better ascertain the circumstances surrounding death.

### Statistical analysis

Data are expressed as means and SDs for quantitative variables and relative frequencies for qualitative variables. In some quantitative variables clinical cut-off values were used. Variables showing a non-linear relationship with the outcome were categorized in tertiles.

The relationship between potential risk factors with CVE was analyzed by bivariate and Fine and Gray competing risk regression models. Chi-squared or McNemar tests were used to compare qualitative variables; the Mann–Whitney test was used for quantitative variables. Significant variables in bivariate analyses and potential confounders were used to develop appropriate multivariate models. Only variables with less than a 5% of missing values were used to build the models. Those variables without a statistically significant contribution but modifying in >10% the value of the coefficients (β) of any of the significant variables when removed from the model were considered confounders and included in the final model.

A competing risks analysis was performed, based on the Fine and Gray model,[25] to estimate the contribution of patients’ characteristics to the cumulative incidence of CVE (both fatal and non fatal). Kidney transplantation and non-cardiovascular death were considered competing events. Different models were fitted for a) all the participants in the study, b) CKD patients not on dialysis and c) patients on dialysis. The model was assessed for interactions of first order using the likelihood ratio test. A statistical significance level of 0.05 was used. A concordance index (C index) was estimated for each right censored survival time fitted, by applying Fine and Gray competing risks models, and assessed at 48 months. The function *cindex*, implemented in the library pec, was used to estimates C indexes for each fitted model. C indexes were used to measure and compare the discriminative power of risk prediction models taking into account different kind of information from vascular imaging (the number of plaques or its recoded version into any plaques yes/no). [26]

## Results

The characteristics and the basal data of the NEFRONA population have been published previously.[11] In the median follow up time of 48.1 months, 216 CVE were reported (Supplemental material, S1 Table). Table 1 displays the bivariate analysis showing factors associated with having a CVE. Patients having an event were predominantly male, older, smokers, diabetics, with a previous diagnostic of hypertension or dyslipidemia, with higher body mass index (BMI), systolic blood pressure (SBP), pulse pressure (PP), cIMT and high sensitivity C reactive protein (hsCRP). They also had a higher number of territories with plaque and a bigger percentage of pathological ankle-brachial index (ABI). In addition, they showed significantly lower levels of total cholesterol, 25(OH) vitamin D and hemoglobin. The hazard ratio of having a CVE increased with the progression of CKD.

**Table 1:** Bivariate analysis of the basal characteristics of the NEFRONA cohort according to the incidence of CVE. Quantitative data are expressed as mean and standard deviation (SD) or median, (p25, p75) depending on the normality of the distribution. Qualitative variables are expressed as N (%). HR: Hazard ratio; CI95%: 95% confidence interval. SBP: systolic blood pressure; PP: pulse pressure; cIMT: intima media thickness; ABI: ankle-brachial index; hsCRP: high sensitivity C-reactive protein. Ref: Reference.

	No event	Event	HR (CI95%)	p
	N = 2788	N = 216		
Sex				
Men	1656 (59.4%)	150 (69.4%)	Ref.	Ref.
Women	1132 (40.6%)	66 (30.6%)	0.63 (0.47;0.85)	0.002
Age (years)	59.5 (48.0;67.0)	65.0 (58.0;70.0)	1.04 (1.03;1.05)	<0.001
BMI (kg/m^2^)	27.7 (24.7;31.1)	29.1 (25.8;32.2)	1.03 (1.00;1.05)	0.027
Smoking				
Never	1221 (43.8%)	75 (34.7%)	Ref.	Ref.
Current/former	1567 (56.2%)	141 (65.3%)	1.47 (1.11;1.95)	0.007
Diabetes				
No	2199 (78.9%)	124 (57.4%)	Ref.	Ref.
Yes	589 (21.1%)	92 (42.6%)	2.61 (1.99;3.41)	<0.001
Hypertension				
No	564 (20.2%)	15 (6.94%)	Ref.	Ref.
Yes	2224 (79.8%)	201 (93.1%)	3.56 (2.11;6.02)	<0.001
Dyslipidemia				
No	1134 (40.7%)	59 (27.3%)	Ref.	Ref.
Yes	1654 (59.3%)	157 (72.7%)	1.70 (1.26;2.29)	0.001
SBP (mmHg)	139 (126;154)	148 (132;163)	1.01 (1.01;1.02)	<0.001
PP (mmHg)	56.0 (47.0;70.0)	66.0 (53.0;80.0)	1.02 (1.01;1.03)	<0.001
cIMT (mm)	0.84 (0.65;1.07)	1.07 (0.88;1.27)	11.2 (6.83;18.3)	0.000
Pathological ABI				
No	2094 (75.7%)	119 (55.1%)	Ref.	Ref.
Yes	671 (24.3%)	97 (44.9%)	2.56 (1.96;3.35)	<0.001
Plaque at baseline				
No	980 (35.2%)	21 (9.7%)	Ref.	Ref.
Yes	1808 (64.8%)	195 (90.3%)	4.80(3.06;7.53)	0.000
Number of territories with plaque	1.00 (0.00;3.00)	3.00 (2.00;6.00)	1.28 (1.22;1.34)	0.000
CKD Stage				
Control	546 (19.6%)	13 (6.02%)	Ref.	Ref.
CKD3	881 (31.6%)	69 (31.9%)	3.28 (1.81;5.93)	<0.001
CKD4-5	739 (26.5%)	68 (31.5%)	4.25 (2.35;7.69)	<0.001
Dialysis	622 (22.3%)	66 (30.6%)	8.34 (4.59;15.2)	<0.001
Total Cholesterol (mg/dl)	181 (156;208)	172 (142;209)	1.00 (0.99;1.00)	0.049
Hemoglobin (gr/dl)	13.0 (11.9;14.4)	12.4 (11.4;13.9)	0.82 (0.76;0.88)	<0.001
Score	1.00 (0.00;3.00)	2.00 (1.00;4.25)	1.16 (1.11;1.21)	<0.001
hsCRP (mg/mL)	1.87 (0.90;4.17)	2.57 (1.25;6.78)	1.01 (1.01;1.02)	0.002
25OHD (ng/ml)	15.7 (11.8;20.3)	13.3 (9.66;17.9)	0.94 (0.92;0.96)	<0.001
1,25(OH)_2_D tertiles (pg/ml)				
(0.1, 11.4)	869 (32.3%)	96 (46.6%)	Ref.	Ref.
(11.4, 22.6)	891 (33.2%)	73 (35.4%)	1.32 (0.92;1.91)	0.137
(22.6,132.2)	927 (34.5%)	37 (18.0%)	1.36 (0.94;1.99)	0.106
Antihypertensive treatment				
No	630 (22.6%)	25 (11.6%)	Ref.	Ref.
Yes	2158 (77.4%)	191 (88.4%)	2.20 (1.45;3.34)	<0.001
Treatment with statins				
No	1350 (48.4%)	80 (37.0%)	Ref.	Ref.
Yes	1438 (51.6%)	136 (63%)	1.54 (1.17;2.03)	0.002

**Fig 1:**
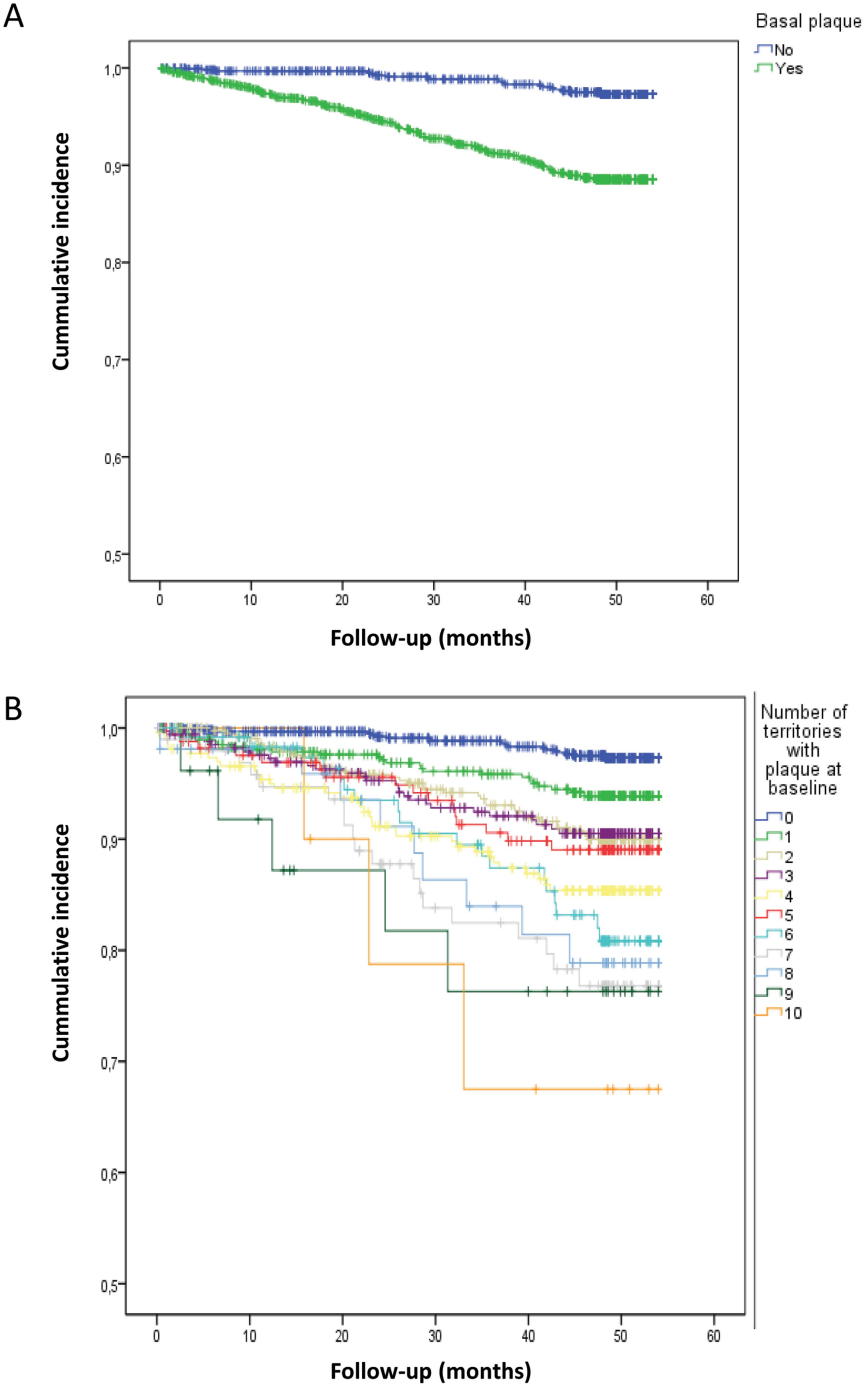
Kaplan-Meier curve showing the unadjusted incidence of CVE along the follow up time (months) depending on (A) the presence or absence of atheroma plaque (p<0.000 Mantel-Haenzel) or (B) the number of arterial territories with atheroma plaque (p<0.000 Mantel-Haenzel linear trend).

In Fig 1 we represent the Kaplan Meier curves showing the time free from cardiovascular events depending on the presence of plaque or the number of territories with plaque. The curves show a significant decrease of survival when patients had at least one territory with plaque (Fig 1A), which is also proportional to the increase in the number of territories with plaque (Fig 1B).

### General model for CKD and non-CKD

**Fig 2:**
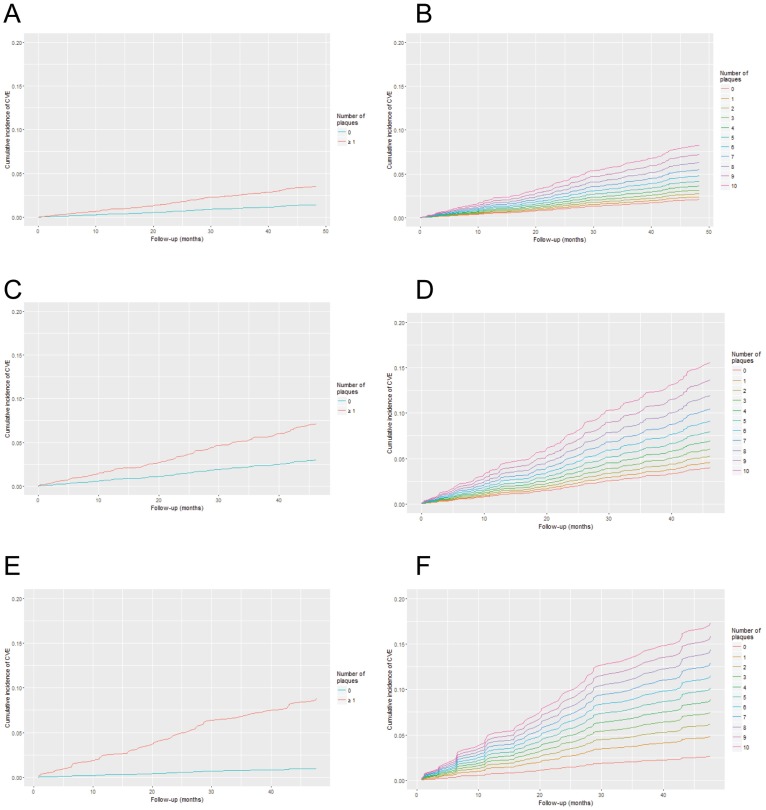
Adjusted cumulative incidence of CVE along the follow up time (months) depending on the presence of atheroma plaque (A, C, and E) or the number of territories with atheroma plaque (B, D and F) in the whole cohort (A, B), CKD patients not on dialysis (C, D) or dialysis patients (E, F). The cumulative incidence was estimated based on the corresponding models provided in Tables 2, 4 and 6.

The competing risks analysis of the incidence of CVE, with death by other causes and kidney transplantation as competing events, is shown in Table 2. During the follow up period, 588 patients underwent a kidney transplant and 110 died by non-cardiovascular causes. The patients' characteristics that predicted a higher cumulative incidence of CVE were being male, a CKD patient (showing an increased risk in more advanced CKD stages), total Cholesterol levels > 240 vs. <200 mg/d, and lower levels of 25(OH) vitamin D. Furthermore, an interaction of age and diabetes was detected. The information of plaque presence at baseline resulted also statistically significant (model 1). When plaque presence was replaced by the number of territories with plaque (model 2) the C index increased. The adjusted cumulative incidence of the population according to the presence of plaque or the number of territories with plaques is depicted in Fig 2A and 2B. The interaction between age and diabetes persisted. Thus, the cumulative incidence of CVE was positively associated with age in non-diabetic patients. In contrast, age did not seem to be a risk factor in diabetic patients, which had a higher risk from early ages (Supplemental material, S1 Fig).

**Table 2:** Multivariate competing risk regression to model the incidence of CVE. Results are expressed as Hazard Ratios (HR; *Exponential β for independent variables with interactions) and 95% Confidence interval (95% CI). The variables introduced to build multivariate models were all the significant variables listed in Table 1 plus any potential confounder.

	Model 1		Model 2	
	HR*	p	HR*	p
Sex (women vs. men)	0.70 (0.52–0.94)	0.017	0.78 (0.58–1.07)	0.12
Diabetes	21.2 (3.83–118)*	<0.001	24.8 (4.54–136)*	<0.001
Age (years)	1.04 (1.02–1.06)*	<0.001	1.04 (1.02–1.06)*	<0.001
Plaque presence	2.50 (1.50–4.17)	<0.001		
Number of territories with plaque (square root)			1.64 (1.35–2.00)	<0.001
CKD3	1.79 (0.98–3.25)	0.058	1.73 (0.95–3.15)	0.074
CKD4-5	2.27 (1.24–4.15)	0.008	2.11 (1.16–3.85)	0.015
Dialysis	3.23 (1.76–5.93)	<0.001	2.86 (1.56–5.25)	<0.001
25(OH)D	0.97 (0.94–0.99)	0.008	0.97 (0.94–0.99)	0.008
Total Cholesterol 200–240	1.05 (0.74–1.49)	0.780	1.03 (0.73–1.46)	0.860
Total Cholesterol>240	2.07 (1.33–3.23)	0.001	1.93 (1.24–3.00)	0.003
Diabetes:Age	0.96 (0.94–0.99)*	0.007	0.96 (0.93–0.99)*	0.001
C-index(4 years)	74.2		74.9	

In order to determine the contribution of specific variables associated with CKD that influence the incidence of CVE, additional regression analysis were performed in CKD patients divided depending on whether or not they were on dialysis.

### CKD patients not on dialysis

A bivariate analysis of the characteristics associated with CVE in patients with CKD without dialysis is shown in Table 3. The competing risks regression analysis showed that all the significant characteristics of the overall cohort plus potassium blood levels > 5.2 mEq/L were associated with a higher cumulative incidence of CVE (Table 4, model 1). Sex and glomerular filtration rate were not significant but it were kept in the model as confounding factors. Again, best C-index was achieved when the number of territories with plaque was included instead of plaque presence/absence (model 2). The adjusted cumulative incidence according to plaque presence or the number of territories with plaque is represented in Fig 2C and 2D. The interaction of age with diabetes was also present in the final model (Supplemental material, S2 Fig). The adjusted effect of potassium is depicted in S3 Fig.

**Table 3:** Bivariate analysis of the basal characteristics of the CKD patients not on dialysis in the NEFRONA cohort according to the incidence of CVE. Quantitative data are expressed as mean and standard deviation (SD) or median, (p25, p75) depending on the normality of the distribution. Qualitative variables are expressed as N (%). HR: Hazard ratio; CI95%: 95% confidence interval. SBP: systolic blood pressure; PP: pulse pressure; cIMT: intima media thickness; ABI: ankle-brachial index; hsCRP: high sensitivity C-reactive protein. Ref: Reference.

	No event	Event	HR (CI95%)	p
	N = 1620	N = 137		
Sex				
Men	999 (62.5%)	100 (73.0%)	Ref.	Ref.
Women	658 (37.5%)	621 (38.3%)	0.59 (0.41;0.86)	0.006
Age (years)	63.0 (53.0,69.0)	66.0 (61.0;70.0)	1.04 (1.02;1.06)	<0.001
BMI (kg/m^2^)	28.3 (25.4;31.9)	29.4 (26.4;32.9)	1.04 (1.01;1.07)	0.008
Smoking				
Never	725 (44.8%)	46 (33.6%)	Ref.	Ref.
Current/former	895 (55.2%)	91 (66.4%)	1.59 (1.11;2.26)	0.011
Diabetes				
No	1186 (73.2%)	74 (54.0%)	Ref.	Ref.
Yes	434 (26.3%)	63 (46.0%)	2.26 (1.61;3.16)	<0.01
Hypertension				
No	123 (7.59%)	4 (2.92%)	Ref.	Ref.
Yes	1497 (92.4%)	133 (97.1%)	2.62 (0.97;7.08)	0.058
Dyslipidemia				
No	484 (29.9%)	26 (19.0%)	Ref.	Ref.
Yes	1136 (70.1%)	111 (81.0%)	1.73 (1.13;2.65)	0.012
SBP (mmHg)	142 (129;157)	154 (135;166)	1.02 (1.01;1.02)	<0.001
PP (mmHg)	59.0 (49.0;72.0)	71.0 (56.0;81.0)	1.02 (1.01;1.03)	<0.001
cIMT (mm)	0.87 (0.67;1.10)	1.08 (0.88;1.29)	9.31 (5.00;17.3)	<0.001
Pathological ABI				
No	1201 (74.7%)	75 (54.7%)	Ref.	Ref.
Yes	406 (25.3%)	62 (45.3%)	2.37 (1.69;3.32)	<0.001
Plaque at baseline				
No	517 (31.9%)	13 (9.5%)	Ref.	Ref.
Yes	1103 (68.1%)	124 (90.5%)	4.21 (2.38;7.46)	0.000
Number of territories with plaque	2.00 (0.00; 4.00)	3.00 (2.00;6.00)	1.24 (1.17;1.32)	<0.001
eGFR (MDRD4)	31.8 (21.2;44.4)	30.1 (19.6;42.6)	0.99 (0.97;1.00)	0.035
Total Cholesterol (mg/dl)				
<200	1109 (69.3%)	89 (65.4%)	Ref.	
200–240	373 (23.3%)	33 (24.3%)	1.07 (0.72;1.60)	0.732
> = 240	119 (7.43%)	14 (10.3%)	1.43 (0.81;2.51)	0.215
Triglycerides (mg/dl)	125 (92.0;174)	146 (106;184)	1.00 (1.00;1.00)	0.031
Calcium (mg/dl)	9.40 (9.10;9.77)	9.40 (8.91;9.70)	0.73 (0.54;1.00)	0.053
Phosphate (mg/dl)	3.60 (3.20;4.11)	3.78 (3.30;4.10)	1.21 (0.98;1.49)	0.079
Sodium (mEq/l)	141 (139;143)	141 (139;142)	0.98 (0.92;1.04)	0.449
Potasium (mEq/l)				
< = 5.2	1111 (69.8%)	79 (58.1%)	Ref.	Ref.
>5.2	481 (30.2%)	57 (41.9%)	1.69 (1.20;2.38)	0.003
Hemoglobin (gr/dl)	13.0 (12.0;14.3)	12.8 (11.8;14.2)	0.94 (0.85;1.04)	0.211
Score	2.00 (1.00;4.00)	3.00 (2.00;5.00)	1.17 (1.11;1.23)	<0.001
hsCRP (mg/mL)	1.87 (0.92;4.00)	2.88 (1.41;7.08)	1.02 (1.01;1.03)	<0.001
25OHD (ng/ml)	15.5 (11.8;19.8)	14.1 (9.72;17.6)	0.94 (0.92;0.97)	<0.001
1,25(OH)_2_D tertiles (pg/ml)				
(0.1, 13.7)	505 (32.5%)	56 (43.8%)	Ref.	Ref.
(13.7, 21.5)	519 (33.4%)	42 (32.8%)	0.70 (0.47;1.04)	0.079
(21.5,89.7)	531 (34.1%)	30 (23.4%)	0.49 (0.31;0.76)	0.001

**Table 4:** Multivariate competing risk regression to model the incidence of CVE. Results are expressed as Hazard Ratios (HR; *Exponential β for independent variables with interactions) and 95% Confidence interval (95% CI). The variables introduced to build multivariate models were all the significant variables listed in Table 3 plus any potential confounder.

	Model 1		Model 2	
	HR*	p	HR*	p
Sex (women vs. men)	0.64 (0.43–0.94)	0.023	0.72 (0.48–1.08)	0.120
Diabetes	17.5 (1.47–209)*	0.024	22.6* (2.00–255)*	0.012
Age (years)	1.05 (1.02–1.08)*	0.003	1.04* (1.01–1.07)*	0.005
Plaque presence	2.43 (1.26–4.69)	0.008		
Number of territories with plaque (square root)			1.15 (1.07–1.25)	<0.001
eGFR (MDRD4)	0.99 (0.98–1.00)	0.096	0.99 (0.98–1.00)	0.15
Potassium >5.2 mEq/L	1.50 (1.03–2.18)	0.036	1.54 (1.06–2.25)	0.024
25(OH)D	0.92 (0.92–0.98)	0.003	0.95 (0.92–0.98)	0.003
Total Cholesterol 200–240	1.25 (0.84–1.88)	0.27	1.25 (0.83–1.87)	0.28
Total Cholesterol>240	1.90 (1.075–3.36)	0.027	1.77 (1.01–3.12)	0.047
Diabetes:Age	0.97 (0.93–1.00)*	0.072	0.96 (0.93–0.99)*	0.036
C-index (4 years)	72.2		72.7	

### CKD patients on dialysis

The bivariate analysis of the characteristics associated with CVE in patients in dialysis is shown in Table 5. The characteristics associated with a higher cumulative incidence of CVE in the multivariate analysis were older age, longer time on dialysis, being diabetic, cholesterol levels > 240 vs <200 mg/dL, higher phosphorous levels and the presence of atheroma plaque (Table 6, model 1). Age and sex did not show statistical significance but were kept in the model as confounders. When plaque extent was used instead of plaque presence, the C index decreased (model 2). The adjusted cumulative incidence according to the presence or number of plaques is represented in Fig 2E and 2F. In S4 Fig (Supplemental material) we represent the adjusted effect of phosphate levels.

**Table 5:** Bivariate analysis of the basal characteristics of the dialysis patients in the NEFRONA cohort according to the incidence of CVE. Quantitative data are expressed as mean and standard deviation (SD) or median, (p25, p75) depending on the normality of the distribution. Qualitative variables are expressed as N (%). HR: Hazard ratio; CI95%: 95% confidence interval. SBP: systolic blood pressure; PP: pulse pressure; cIMT: intima media thickness; ABI: ankle-brachial index; hsCRP: high sensitivity C-reactive protein. Ref: Reference.

	No event	Event	HR (CI95%)	p
	N = 622	N = 66		
Sex				
Men	366 (58.8%)	43 (65.2%)	Ref.	Ref.
Women	256 (41.2%)	23 (34.8%)	0.70 (0.42;1.16)	0.164
Age (years)	53.0 (42.0;64.0)	62.0 (52.2;68.0)	1.03 (1.01;1.05)	0.002
BMI (kg/m^2^)	25.7 (23.0;28.9)	26.7 (23.7;30.2)	1.02 (0.97;1.06)	0.452
Smoking				
Never	278 (44.7%)	25 (37.9%)	Ref.	Ref.
Current/former	344 (55.3%)	41 (62.1%)	1.49 (0.90;2.45)	0.118
Diabetes				
No	525 (84.4%)	39 (59.1%)	Ref.	Ref.
Yes	97 (15.6%)	27 (40.9%)	3.41 (2.09;5.57)	<0.001
Hypertension				
No	85 (13.7%)	6 (9.09%)	Ref.	Ref.
Yes	537 (86.3%)	60 (90.9%)	1.58 (0.68;3.65)	0.287
Dyslipidemia				
No	293 (47.1%)	28 (42.4%)	Ref.	Ref.
Yes	329 (52.9%)	38 (57.6%)	1.03 (0.63;1.69)	0.892
SBP (mmHg)	137 (123;153)	141 (120;158)	1.00 (0.99;1.01)	0.943
PP (mmHg)	56.0 (45.0;70.0)	60.0 (44.2;75.0)	1.01 (0.99;1.02)	0.395
cIMT (mm)	0.69 (0.59;0.84	0.75 (0.67;1.08)	4.33 (1.94;9.70)	<0.001
Pathological ABI				
No	416 (67.9%)	32 (48.5%)	Ref.	Ref.
Yes	197 (32.1%)	34 (51.5%)	1.92 (1.18;3.11)	0.008
Plaque at baseline				
No	205 (33%)	2 (3%)	Ref.	Ref.
Yes	417 (67%)	64 (97%)	12.44 (3.05;50.84)	0.000
Number of territories with plaque	2.00 (0.00;4.00)	4.00 (2.00;5.00)	1.24 (1.14;1.35)	<0.001
Time on dialysis (months)	18.0 (7.85;34.1)	23.9 (10.7;42.3)	1.00 (1.00;1.01)	0.351
Total Cholesterol (mg/dl)				
<200	496 (82.3%)	54 (83.1%)	Ref.	Ref.
200–240	87 (14.4%)	4 (6.15%)	0.44 (0.16;1.22)	0.116
> = 240	20 (3.32%)	7 (10.8%)	2.32 (1.06;5.11)	0.036
Triglycerides (mg/dl)	123 (88.5;168)	121 (93.0;178)	1.00 (1.00;1.00)	0.995
Calcium (mg/dl)	9.10 (8.60;9.50)	9.00 (8.72;9.52)	1.13 (0.80;1.58)	0.485
Phosphate (mg/dl)	4.80 (4.00;5.57)	5.30 (4.40;6.16)	1.24 (1.06;1.47)	0.009
Sodium (mEq/l)	139 (137;141)	138 (136;140)	0.93 (0.86;1.00)	0.059
Potasium (mEq/l)				
< = 5.2	378 (61.5%)	32 (49.2%)	Ref.	Ref.
>5.2	237 (38.5%)	33 (50.8%)	1.33(0.82;2.17)	0.246
Hemoglobin (gr/dl)	11.9 (11.1;12.8)	11.6 (10.6;12.5)	0.91 (0.77;1.08)	0.304
Score	1.00 (0.00;2.00)	2.00 (1.00;3.00)	1.13 (1.05;1.22)	0.001
hsCRP (mg/mL)	2.24 (0.97;5.49)	2.42 (1.22;6.82)	0.99 (0.97;1.02)	0.521
25OHD (ng/ml)	13.7 (10.3;18.5)	12.4 (9.25;16.7)	0.97 (0.93;1.01)	0.126
1,25(OH)_2_D tertiles (pg/ml)				
(0.1, 4.56)	199 (32.9%)	25 (38.5%)	Ref.	Ref.
(4.56, 7.63)	200 (33.1%)	22 (33.8%)	0.86 (0.49;1.53)	0.610
(7.63, 48.04)	205 (33.9%)	18 (27.7%)	0.61 (0.33;1.12)	0.112

**Table 6:** Multivariate competing risk regression to model the incidence of CVE. Results are expressed as Hazard Ratios (HR) and 95% confidence interval. The variables introduced to build multivariate models were all the significant variables listed in Table 5 plus any potential confounder.

	Model 1		Model 2	
	HR	p	HR	p
Sex (women vs. men)	0.85 (0.50–1.44)	0.540	0.90 (0.53–1.54)	0.700
Diabetes	2.73 (1.63–4.56)	<0.001	2.65 (1.57–4.48)	<0.001
Age (years)	1.02 (0.99–1.04)	0.150	1.02 (0.99–1.04)	0.260
Plaque presence	9.23 (2.03–41.9)	0.004		
Number of territories with plaque (square root)			1.86 (1.34–2.58)	<0.001
Months in dialysis (logarithmic scale)	1.39 (1.09–1.76)	0.007	1.38 (1.09–1.75)	0.007
Phosphate (mg/dL)	1.30 (1.11–1.53)	0.001	1.29 (1.09–1.51)	0.003
Total Cholesterol 200–240	0.54 (0.19–1.49)	0.230	0.53 (0.19–1.49)	0.230
Total Cholesterol>240	3.86 (1.76–8.48)	<0.001	3.29 (1.46–7.42)	0.004
C-index (4 years)	78.7		78.3	

## Discussion

In the present work, we demonstrate for the first time that the determination of subclinical atheromatosis in CKD patients not on dialysis strongly predicts the incidence of cardiovascular events. Furthermore, the quantification of the atheroma extent in 10 arterial territories using arterial ultrasound is a better predictor of cardiovascular events than plaque presence in this population. Our data also confirm previous results showing that in dialysis patients, subclinical atheromatosis is also a predictor of cardiovascular events. In this kind of patients, the determination of plaque extent is not a better predictor that the presence/absence of atheroma plaque. The multivariate regression models show that plaque information has some of the strongest HR in the model. Thus, in the total population, plaque presence increases the risk 2.5 times and induces a 15% increase risk per each territory with plaque, independently of other variables like CKD stage, age or diabetes. In the CKD population not in dialysis the weight of the variables is similar to the previous values but in the dialysis population, the presence of plaque multiplies the risk of having a CVE by 9. Furthermore, having a new territory with plaque increases the risk of having an event by 86%, although this relationship is not linear and the weight of every new territory with plaque declines successively. Therefore, the results endorse the role of atheromatosis in cardiovascular mortality in CKD and confirm the use of arterial ultrasound as a tool in cardiovascular risk prediction in renal patients.

The use of ultrasonography for the detection of subclinical atherosclerosis requires no ionizing radiation, is highly reproducible, and can typically be done in an office setting. Furthermore, numerous studies involving diverse populations have demonstrated that atherosclerotic imaging more accurately identifies individual risk as compared to risk scores.[27, 28] This could be of particular interest in special populations like CKD, in which traditional risk prediction formulas are remarkably inaccurate.[6] Several studies have analyzed the potential predictive effect of carotid ultrasound in cardiovascular events, but many excluded the presence of plaques and only incorporated in the analysis the ccIMT values.[12, 29] However, when plaque presence is included, the prediction capability of the resulting formula increases significantly.[27, 30, 31]

To enhance the predictive power of plaque screening for CVE beyond plaque presence, quantifying the carotid plaque extent has been developed. Carotid plaque extent has been measured previously either as total plaque area by 2-dimensional ultrasound or total plaque volume by 3-dimensional ultrasound. These techniques are not regularly available in the clinical settings making difficult to clinicians its use in their daily practice. In our study, we assessed plaque extent by identifying the number of territories that presented plaque, an approach more reproducible in the daily clinical settings. Additionally, we also explored the femoral area, identifying individuals with plaques exclusively in the femoral arteries, which can account for a significant percentage in the renal population.[11] In our study, cIMT values were not statistically significant predicting the occurrence of a cardiovascular event. Our results agree with previous reports in non-CKD populations showing that cIMT is a weak predictor of cardiovascular risk [32] and that plaque extent can strongly predict CVE.[33, 34] Furthermore, the inclusion of plaque extent increased the C-index.

The analysis of the whole NEFRONA population confirmed that being a CKD patient conferred a higher CVE risk, with increased HR in more advanced stages of the disease. Furthermore, and agreeing with previous studies[35–37] the results also confirm that low vitamin D levels are predictors of a CVE. In addition, the results also confirm that being diabetic is a risk factor for having a CVE[38] and that it eliminates the effect of age. Finally having total cholesterol levels over 240 mg/dl also predicted the occurrence of a CVE.

To identify specific factors related to CKD, we built two additional models in CKD patients depending on whether or not they were undergoing dialysis. The model for CKD patients not on dialysis showed that, additionally to all the factors identified in the general model, levels of K^+^ over 5.2 mEq/L can predict the occurrence of a CVE. Very high serum K^+^ levels predisposes patients to ventricular arrhythmia and sudden death.[39] However, the effect of small increases in serum potassium on CVE in the renal population has not been thoroughly studied.[40] Our results agree with a very recent paper of Luo[41] showing that levels of K^+^ over 5.5 mEq/L significantly increased mortality risk.

The analysis in the dialysis population showed that the information about plaque, did not increase the predictive power of the model, compared with the determination of plaque presence. In this case, plaque extent information did not improve risk prediction. Therefore, in dialysis patients, the determination of plaque presence/absence could be enough to predict CVE risk. The presence of diabetes and higher levels of total cholesterol also displayed a significant effect. In ESRD populations, the relationship of serum cholesterol levels with CVD events and all-cause mortality is inconsistent and often paradoxical.[42–44] In fact, cholesterol levels usually decrease in more advanced CKD stages.[11] This apparent inconsistency has been explained by the confounding effect of the commonly present malnutrition and inflammation.[45] In our cohort, higher levels of total cholesterol were predicting the occurrence of a CVE. This effect was also present in dialysis patients, in which total cholesterol levels over 240 mg/dL showed a strong weight. This could be explained by the fact that NEFRONA patients, in contrast with many other dialysis cohorts, were free from any previous cardiovascular disease and with an age limit of 75 years old.

In our model, the dialysis vintage also predicted the occurrence of a CVE, so the longer a patient stayed in dialysis, the higher the risk of suffering a CVE, independently of their age. In addition, higher levels of phosphate also increased the risk. Since the first report showing that phosphate levels are associated with mortality in hemodialysis patients[46] the role of phosphate has been widely investigated. Thus, phosphate levels can induce CVE by increasing vascular calcification[47] and FGF23 levels, which have been shown to be associated with cardiovascular events.[48] Furthermore, higher levels of phosphate have been linked to an increase in the presence and the progression of atheroma plaque [49–51], which can account for part of the increased risk observed in our population.

The main strength of this study is the large number of patients with longitudinal observations, which allows us to make predictive associations between multiple factors and CVE and adjust for multiple confounders. Furthermore, the follow up period of 4 years gave us enough number of events to obtain a reliable model. Another strength is that the vascular exploration was performed by the same team and evaluated by a single reader.

Our study has several limitations. First, the NEFRONA study did not have a committee reviewing the CVE, and they were obtained from the clinical history of each patient. However, CVE were confirmed by a telephonic interview. Second, fibroblast growth factor 23 levels were lacking, which could have been mediating the effects of high phosphate in cardiovascular events in dialysis patients. In addition, the large number of dropouts, mainly by renal transplant, might have slightly biased our population towards patients with a worse general health condition.

In conclusion, subclinical atheromatosis strongly affects the occurrence of CVE in CKD patients. The quantification of atheromatosis extent by arterial ultrasound it is a parameter easy to obtain, and can be used in routine clinical practice to the assess CVE risk prediction in CKD patients.

## Supporting information

S1 Fig Interaction age-diabetes in the whole cohort. Adjusted cumulative incidence of CVE in the NEFRONA cohort. A) Effect of age in diabetic patients. B) Effect of age in non-diabetic patients.
(PDF)

S2 Fig Interaction age-diabetes in the CKD patients not in dialysis. Adjusted cumulative incidence of CVE in CKD patients not in dialysis. A) Effect of age in diabetic patients. B) Effect of age in non-diabetic patients.
(PDF)

S3 Fig Effects of potassium levels in the CKD patients not in dialysis. Adjusted cumulative incidence of CVE in CKD patients not in dialysis. Effect of levels of potassium.
(PDF)

S4 Fig Effects of phosphate levels in the CKD patients not in dialysis. Adjusted cumulative incidence of CVE in CKD patients in dialysis. Effect of levels of phosphate.
(PDF)

S1 Table Cardiovascular events registered during the follow-up. (DOCX)
