# Supplementary material for: Factors predicting cardiovascular events in chronic kidney disease patients. Role of subclinical atheromatosis extent assessed by vascular ultrasound
Source: PLoS One. 2017 Oct 18;12(10):e0186665. doi: 10.1371/journal.pone.0186665 (PMC5646852; doi:10.1371/journal.pone.0186665)
Supplement: S2 Fig — Adjusted cumulative incidence of CVE in CKD patients not in dialysis. A) Effect of age in diabetic patients. B) Effect of age in non-diabetic patients. (PDF) [file pone.0186665.s002.pdf]

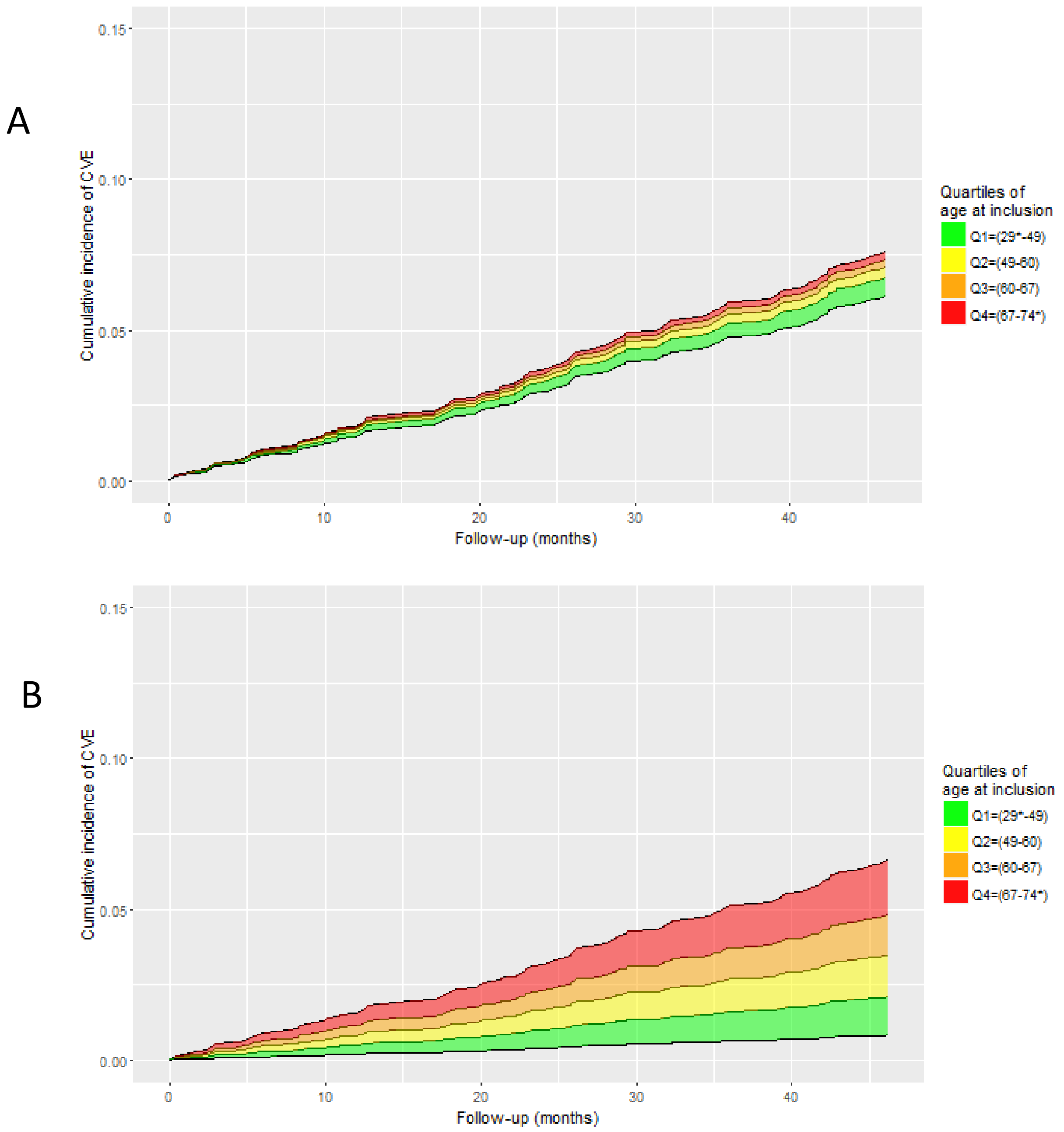

Figure S2: Adjusted cumulative incidence of CVE in CKD patients not in dialysis. A) Effect of age in diabetic patients. B) Effect of age in non-diabetic patients.

Figure S2
