# Supplementary material for: Factors predicting cardiovascular events in chronic kidney disease patients. Role of subclinical atheromatosis extent assessed by vascular ultrasound
Source: PLoS One. 2017 Oct 18;12(10):e0186665. doi: 10.1371/journal.pone.0186665 (PMC5646852; doi:10.1371/journal.pone.0186665)
Supplement: S1 Table — (DOCX) [file pone.0186665.s005.docx]

**Table S1. Cardiovascular events reported in the follow-up period.**

| Event | Number |
| --- | --- |
| Myocardial infarction | 37 |
| Unstable angina | 32 |
| Cerebrovascular accident | 37 |
| PAD or amputation for vascular disease | 25 |
| Transient ischemic attack | 22 |
| Stent/Bypass | 17 |
| Mesenteric infarction | 9 |
| Aortic aneurism | 8 |
| Sudden death | 16 |
| Congestive heart failure | 6 |
| Stroke | 2 |
| Arrhythmia | 1 |
| Other | 4 |
| Total | 216 |
